# Chitinases: expanding the boundaries of knowledge beyond routinized chitin degradation.

**DOI:** 10.1007/s11356-024-33728-6

**Published:** 2024-05-24

**Authors:** John Onolame Unuofin, Olubusola Ayoola Odeniyi, Omolara Sola Majengbasan, Aboi Igwaran, Karabelo MacMillan Moloantoa, Zenzile Peter Khetsha, Samuel Ayodele Iwarere, Michael Olawale Daramola

**Affiliations:** 1 Sustainable Energy and Environment Research Group (SEERG), Department of Chemical Engineering, Faculty of Engineering, Built Environment and Information Technology, University of Pretoria, Private bag X20 Hatfield, Pretoria, 0028 South Africa; 2 Department of Microbiology, University of Ibadan, Ibadan, Nigeria; 3 The Life Science Center Biology, School of Sciences and Technology, Örebro University, 701 82 Örebro, Sweden; 4 Department of Microbiology, School of Life Sciences, College of Agriculture, Engineering and Science, University of Kwazulu Natal, Private Bag X540001, Durban, 4000 South Africa; 5 Department of Agriculture, Central University of Technology, Free State, Private Bag X20539, Bloemfontein, 9300 South Africa

**Keywords:** Chitin, Chitin degradation, Chitinase, Biotechnolgical applications, Biological warfare

## Abstract

Chitinases, enzymes that degrade chitin, have long been studied for their role in various biological processes. They play crucial roles in the moulting process of invertebrates, the digestion of chitinous food, and defense against chitin-bearing pathogens. Additionally, chitinases are involved in physiological functions in crustaceans, such as chitinous food digestion, moulting, and stress response. Moreover, chitinases are universally distributed in organisms from viruses to mammals and have diverse functions including tissue degradation and remodeling, nutrition uptake, pathogen invasion, and immune response regulation. The discovery of these diverse functions expands our understanding of the biological significance and potential applications of chitinases. However, recent research has shown that chitinases possess several other functions beyond just chitin degradation. Their potential as biopesticides, therapeutic agents, and tools for bioremediation underscores their significance in addressing global challenges. More importantly, we noted that they may be applied as bioweapons if ethical regulations regarding production, engineering and application are overlooked.

## Introduction

In the captivating world of enzymology, few enzymes rival the allure and scientific significance of chitinases, owing to their remarkable versatility and largely unexplored potential. These extraordinary biomolecules have long captivated researchers, as they hold the key to unraveling the enigmatic realm of chitin degradation and far beyond. Chitinases, in their multifaceted functionality, have garnered immense attention for their pivotal role in various biological systems, transcending the boundaries of mere chitin breakdown (Mahajan et al. 2023). As we venture into this critical exploration, we embark on a journey that transcends conventional limits, delving into the intricate complexities that define chitinases' remarkable influence on nature's grand tapestry. Chitin, an abundant polysaccharide found ubiquitously in the cell walls of fungi, exoskeletons of arthropods, and other crustaceans, stands as an unyielding fortress, imparting resilience and structural integrity to countless living organisms (Bai et al. 2022). Chitinases, as the vanguard of chitin hydrolysis, were initially studied for their significance in the natural recycling processes, enabling the cyclic flow of nutrients within ecosystems (Kumar et al. 2022; Thakur et al. 2023a). However, in the quest to understand the profound intricacies of these fascinating enzymes, researchers have been astounded by their diverse and intricate roles in a myriad of biological functions. Beyond their traditional role in chitin breakdown, chitinases have emerged as pivotal players in a plethora of biological phenomena. From their participation in plant defense mechanisms against pathogenic invaders to their impact on human health, chitinases have proven to be indispensable components of both innate and adaptive immunity (Jiang et al. 2022; Vaghela et al. 2022; Mahajan et al. 2023). Furthermore, their intriguing involvement in insect metamorphosis and development has opened new avenues of research into the regulation of growth and morphogenesis (Girard et al. 2022). Moreover, recent advances in biotechnology have brought chitinases to the forefront as potential eco-friendly solutions in the management of chitinous waste, contributing to the sustainable future of waste management and resource utilization (Mahajan et al. 2023). Their ability to modify chitin-derived materials has led to exciting possibilities in fields ranging from biomedicine to agriculture and environmental remediation. Yet, as our knowledge about chitinases expands, so does our awareness of the countless mysteries that lie ahead. The intricate mechanisms underlying their catalytic activities, the intricate interplay of their isoforms, and their varied cellular functions continue to challenge our scientific understanding. To unlock their full potential, novel strategies, cutting-edge technologies, and interdisciplinary collaboration are essential. In this critical exploration, we embark on a quest to delve deeper into the profound implications of chitinases in a plethora of biological processes, transcending their traditional role in chitin degradation. As we venture further into uncharted territories, we strive to push the boundaries of knowledge, unearthing the hidden secrets that chitinases hold, and ultimately paving the way for innovative applications across diverse scientific domains.

## Chitin: nuisance and degradation

**Fig. 1:**
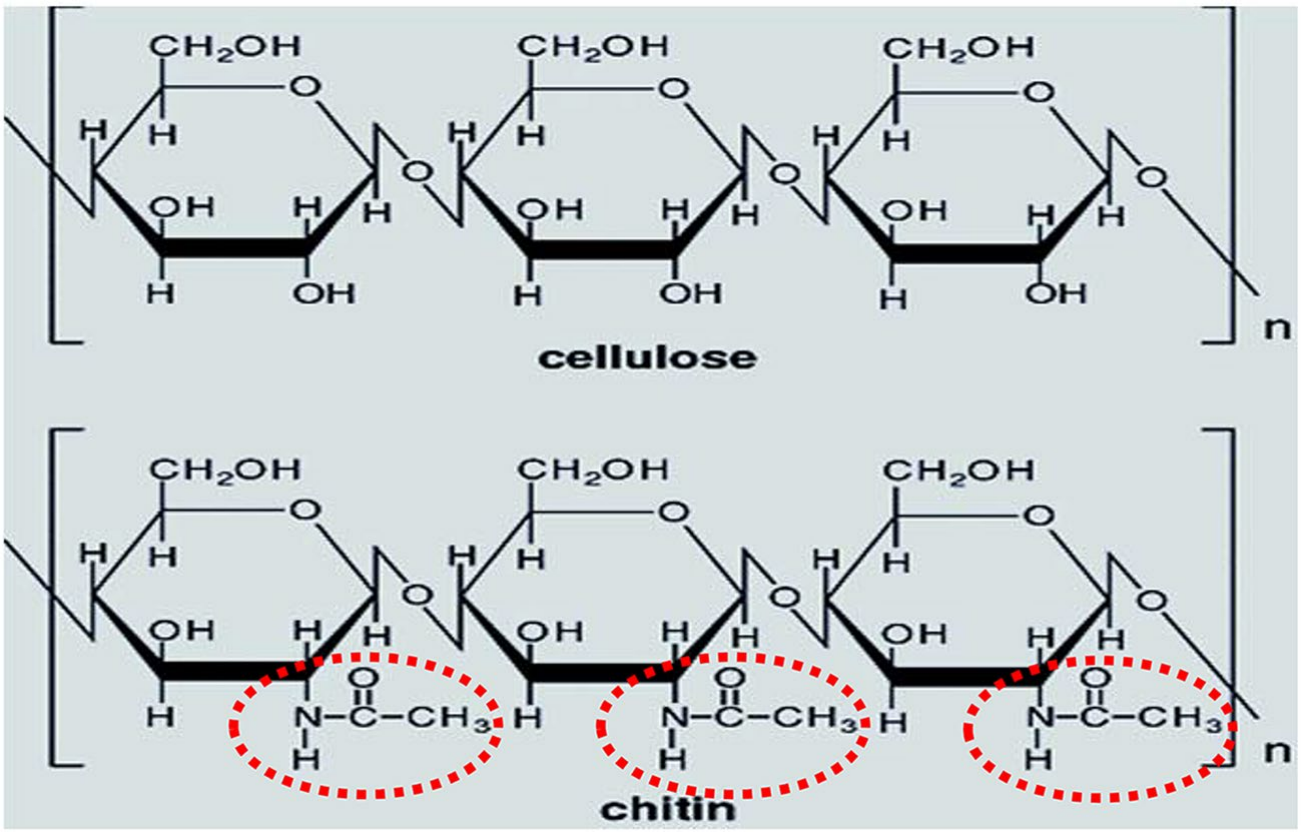
Distinguishing a chitin from cellulose. The N-acetyl group attached to the β-(1,4)-glycosidic linkages is encircled in dotted red (source: this study)

Chitin shares similarity in structure with cellulose in that they are both derivatives of glucose monomers. However, it differs structurally due to the substitution of the hydroxyl group for N-acetyl group at the β-(1,4)-glycosidic linkages (Fig. 1), which confers a more stable, rigid, and stronger scaffold than cellulose (Tabli and Katiyar 2020; Hou et al. 2021; Kobayashi et al. 2023). Being the second most abundant polymer after cellulose, the estimated annual production of chitin from the aquatic biosphere is staggering, ranging from 10^12^ to 10^14^ tonnes, indicating its wide distribution and prevalence as constituents of the anatomical framework of many organisms (Ofem et al. 2017; Rkhaila et al. 2021). However, this abundance is not without consequences, as the excessive accumulation of chitin waste poses significant pollution problems.

**Fig. 2:**
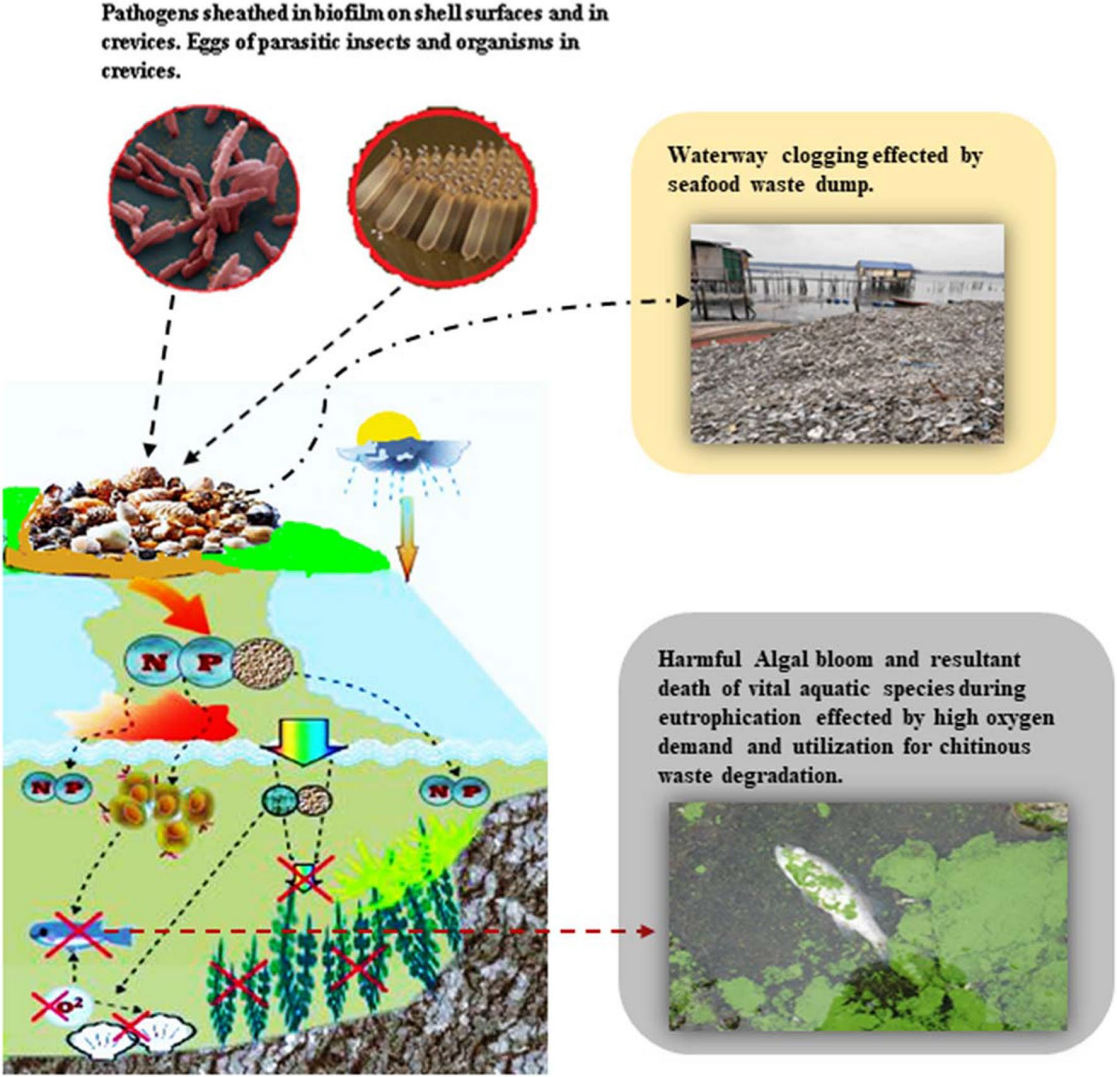
Environmental and public health effects of chitinous waste dumping scenarios. The bottom left pane describes the enrichment of water bodies with nutrients (N: nitrogen, P: phosphorus) and the subsequent depletion of biologically available oxygen (O_2_: molecular oxygen)

The utilization of chitin is not fully optimized, leading to substantial portions being discarded as “waste” after processes like shrimp and crab shell processing or waste from the seafood industry. Alarmingly, no less than 60% of chitin-based residue of seafood and molluscs is discarded without proper management or recycling practices (Yadav et al. 2019; Chakravarty and Edwards 2022). This mismanagement exacerbates pollution issues, as chitin is highly resistant to active degradation in natural environments. Its robust structure, resulting from tough and rigid units of linked N-acetylglucosamine monomers as well as slow breakdown rates pose challenges for certain ecosystems to adapt to influx of chitin waste (Amiri et al. 2022). When stockpiled on soil, chitin of crustacean shells could facilitate land fill waste accumulation, occupying valuable space and causing nuisance. Consequently their composting might emit unpleasant odour and leaching of nutrients to pollute groundwater as well as greenhouse gases, which could contribute substantially to atmospheric warming in the long run (Ngasotter et al. 2023; Topić Popović et al. 2023). In aquatic environments, chitin wastes accumulation can disrupt the delicate balance of ecosystems. In areas with extensive chitin pollution, such as near seafood processing facilities or waste disposal sites, negative effects on marine life are evident (Wani et al. 2023). For instance, as chitin resists degradation, its slow but eventual microbial decomposition consumes substantial oxygen, creating an imbalance in the ecosystem's oxygen-demanding processes. This phenomenon could have far-reaching ecological consequences, such as creating dead zones (oxygen-depleted zones) that are uninhabitable for certain aquatic life, and thereby affecting entire food chains and biodiversity. Conversely, chitin particles may clog waterways due to enhanced and accelerated sedimentation processes and alter the behavior and feeding patterns of aquatic organisms. Moreover, chitin-rich waste may provide favorable conditions for the growth of harmful microorganisms, leading to the deterioration of water quality and posing risks to human health (Fig. 2) (Mathew et al. 2021a, and references therein; Wani et al. 2023). Therefore, addressing chitin pollution requires a multifaceted approach that involves promoting sustainable waste management practices, developing effective recycling technologies, and encouraging the utilization of chitin in various applications. We opine that biotechnological advances may hold the key to finding innovative solutions for chitin waste treatment and recycling, thereby reducing its negative impact on the environment.

**Fig. 3:**
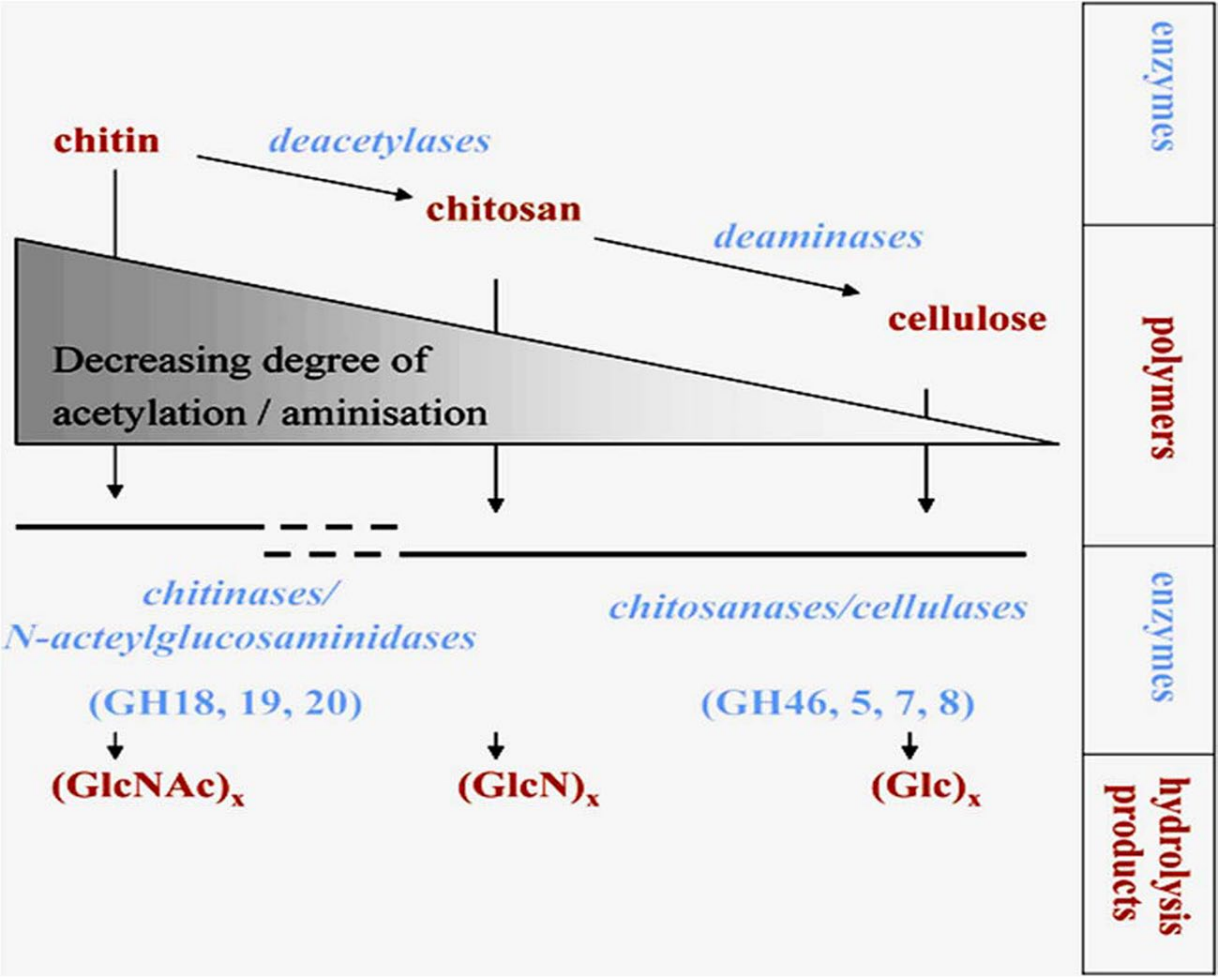
Chitin degradation process (Beier and Bertilsson 2013)

Rhizospheric bacteria in soil utilize chitin from fungi and insects as nitrogen and carbon sources (Singh et al. 2021), while marine habitats demonstrate chitin-enabled nutrient cycling from arthropod shells and other sources (Jahromi and Barzkar 2018). This is facilitated by chitin degradation through anaerobic or aerobic processes, usually involving chitinolytic microorganisms. Chitin degradation is preceded by the hydrolysis of its (1→4)-β glycoside bond, termed chiotinolysis, which involves chitinolytic microbes and enzymes. However, this phenomenon may also be facilitated by other lytic enzymes, such as lytic polysaccharide monooxygenase (LPMO). Beier and Bertilsson (2013) found that microbial growth on chitin may not always lead to its depolymerization, as some microbes can metabolize other substrates, such as atmospheric nitrogen and CO_2_. Chitin can also undergo deacetylation to chitosan or further deamination to form cellulose (Fig. 3) (Bonin et al. 2020). Chitin degradation by bacteria is widespread, playing a vital role in biogeochemical cycles. This process is tightly regulated, with chitin hydrolysis products, N-Acetylglucosamine (GlcNAc) and soluble chitin, acting as inducers of hydrolytic enzymes (Yusuke et al. 2020; Kristie et al. 2021). The expression of chitinases and hydrolytic enzymes is influenced by factors like growth substrates and nutrient regimes (Delpin and Goodman 2009; Beier and Bertilsson 2013), highlighting the array of ecological niches where chitin degradation occurs. Complete lysis of insoluble chitin involves three steps: cleaving the polymer into water-soluble oligomers, splitting the oligomers into dimers, and finally cleaving dimers into monomers (Beier and Bertilsson 2013). Multiple chitinases in an organism enhance substrate utilization through synergistic interactions (Oyeleye and Normi 2018). Various techniques, such as measuring chitin weight loss, ^14^C labeled experiments, fluorogenic substrate analogs, or colorimetric incubation, aid in assessing chitin hydrolysis (Arnold et al. 2020). Temperature is a vital factor influencing chitin degradation rates, with higher temperatures often leading to increased activity (Kuzmina et al. 2020). Chitinolytic bacteria may produce more chitin oligomers than they can utilize, which influences its degradation rates in the environment (Beier and Bertilsson 2013; Rathor and Gupta 2015). Hydrolysis products released in natural environments can serve specific populations and facilitate inter-species feeding, as some bacteria can use N-acetyl glucosamine or glucosamines without chitinolytic activity (Beier and Bertilsson 2013). In aerated soils, hydrolysis products remain in proximity to the enzymatic action site (Robin and Dani 2017).

## Chitinases: sources, function, and microbial production

Chitinases are found in various organisms: mammals, plants, insects, viruses, fungi, and bacteria (Karthik et al. 2017). They have distinct functions, including turnover of cuticles, digestion, cell differentiation, and defense against pathogens. In mammals, chitinases can be true chitinases (breakdown and digest chitin) or protein chitinases (bind to chitin) (Przysucha et al. 2020; Hamid et al. 2013) and can play important roles in mammalian protection against pathogens (Hu et al. 2021). Plant chitinases, classified under glycosyl hydrolases family 19 with molecular weights ranging from 20 to 40 kDa, are activated in response to phytopathogenic attacks, aiding self-defense (Malik 2019; Vaghela et al. 2022). Their secretion is usually tissue-specific, preventing the growth of parasitic fungal hyphae and can enhance protection against pathogenic fungi in plants, under environmental stress (Kumar et al. 2018). Insect chitinases (family GH18) facilitate moulting and transformation from larvae to adults (endochitinases and exochitinases), especially in the removal of old exoskeletons (Chen and Yang 2020). Their molecular weights vary between 40 and 85 kDA and are expressed in various insect orders (Karthik et al. 2017). Viral chitinases (GH18 family) help infect or discharge viruses by weakening host barriers (Berini et al. 2018). Fungal chitinases (family 18) assist in cell wall formation and hyphal processes. They are divided into groups A, B, and C and are secreted by fungi like *Aspergillus, Penicillium, Trichoderma*, and *Neurospora* (Goughenour et al. 2020; Langner and Gohre 2016). Here, the entomopathogenic and mycoparasitic strains, such as *Trichoderma* and *Neurospora* possess multiple extracellular glycosyl hydrolase 18 chitinolytic machinery, which directly penetrate the host’s defense system during attack (Berini et al. 2018). Bacterial chitinases (GH18 and GH19 families) have subfamilies A, B, and C, and their sizes range from 20 to 60 kDa (Juárez-Hernández et al. 2019). They play a crucial role in the degradation of chitin in various biogeochemical ecosystems (Beier and Bertilsson 2013). Bacteria in marine habitats and genera like *Bacillus* produce chitinases involved in the degradation of recalcitrant materials in the ocean (Dhole et al. 2021). Purification and characterization studies infer that they possess a broad range of pH, temperature and isoelectric points, depending on their isolation points (Rathore and Gupta 2015).

The profusion of bacteria in soil systems has been linked to the rate of chitin hydrolysis, which is dependent on pH, temperature as well as the succession of the degradation process. In corroboration, in situ research and plating demonstrated that bacteria are major mediators in chitin degradation and can extend this phenomenon to various chitin analogues in nature (Beier and Bertilsson 2013; Juárez-Hernández et al. 2019). In an ex situ scenario, microbial chitinase can be produced through submerged fermentation (batch, fed-batch, and biphasic cell systems) or solid-state fermentation. Submerged fermentation allows easy enzyme recovery and process control, while solid-state fermentation is advantageous for easy operation and cost-effective raw materials and concentrated enzyme recovery. However, solid-state fermentation faces challenges with substrate sterilization, culture purity, pH and temperature control as well as a prolonged fermentation process (Karthik et al. 2017). Due to the canonically inducible nature of microbial chitinases, chitin availability is sacrosanct for their enhanced yield. In this regard, colloidal chitin is reported to be the most effective inducer, albeit chitinous substrates of diverse organisms (prawn, crab and shrimp waste), as well as agro-industrial residues (rice bran, wheat bran etc.), might markedly influence chitinase synthesis (Karthik et al. 2017; Wahab and Esawy 2022). Apart from chitin-induced enzymatic secretions, various physiochemical parameters, such as media components, pH, temperature, aeration, carbon, and nitrogen sources, influence chitinase production. Interestingly, the addition of ancillary carbon sources, especially simple sugar, such as maltose, glucose, sucrose, lactose, and arabinose alongside colloidal chitin can have varying effects on production (Meena et al. 2015; Atheena et al. 2024). Organic nitrogen sources like peptone, corn steep liquor, malt extract, and yeast extract, as well as inorganic sources like nitrates and ammonium salts, positively influence chitinase production (Singh et al. 2020). Incubation temperature and pH play crucial roles, typically achieving the highest production at near-neutral pH (6.0-8.0) and mesophilic temperatures (25-35°C) (He et al. 2020; Singh et al. 2021). Factors like cell membrane porosity, surfactant concentrations, and the addition of metal ions can also affect chitinase production. Prolonged incubation periods may reduce chitinase production due to nutrient depletion and the production of inhibitory chemicals (Karthik et al. 2017).

## Microbial chitinases: physicochemical and molecular profile

**Fig. 4:**
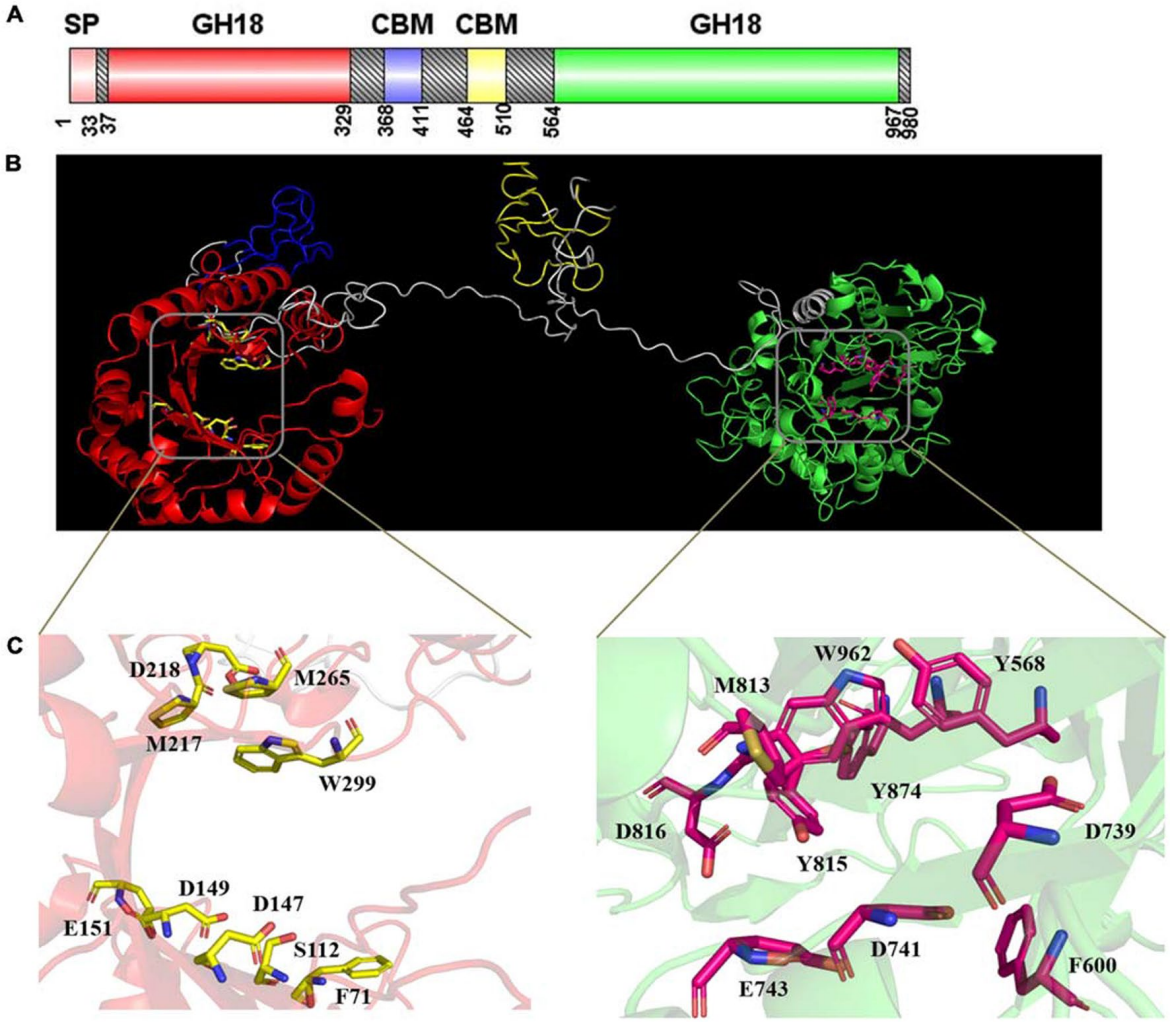
Structural features of *Cm*Chi3 showing: **(A)** a putative signal sequence, two GH18 catalytic domains, and two carbohydrate-binding modules (CBMs). **(B)** forecasted 3D structure where red indicates Glyco_18 domain; blue indicates CBM; yellow indicates CBM; green indicates Glyco_18 domain; gray indicates the unknown region. **(C)** The active sites, where D147, D149, and E151 were the active residues of the N-terminal catalytic domain; whereas D739, D741, and E743 were the active residues of the C-terminal catalytic domain. [Reproduced from Wang et al. (2022) (CC BY 4.0)]

Although all microbial chitinases possess the ability to facilitate the catalytic breakdown and transformation of chitinous substrates, they vary in their primary sequences, three-dimensional structures, expression patterns, physicochemical characteristics and catalytic mechanisms. Understanding the molecular and catalytic characteristics of chitinases will enable researchers to engineer and optimize these enzymes for specific applications. Studies have revealed that chitinases are encoded by a wide range of genes across various organisms, including bacteria, fungi, plants, and animals (Oyeleye and Normi 2018; Kim et al. 2021; Kumar et al. 2022; Thakur et al. 2023b). Moreover, molecular cloning and sequencing of these genes have allowed researchers to identify conserved catalytic domains and motifs that are characteristic of chitinases, such as the glycoside hydrolase families 18 and 19 (GH18 and GH19) domain. Genomic and transcriptomic analyses have also led to the discovery of novel chitinases with unique characteristics, such as interesting substrate specificities and enzymatic activities brought about by variations in amino acid sequences (Renaud et al. 2023). Chitinases exhibit diverse physicochemical properties, including molecular weight, isoelectric point (pI), thermal stability, and pH optima. For instance, most chitinases have been observed to exhibit optimal catalytic activity within the pH range of 3 – 10 and a temperature optima of 25–70°C (Thakur et al. 2023b). However, psychrotolerant variants that retain 90% residual activity at temperature of 0–20°C have been discovered likewise (Chen et al. 2024). The molecular weight of chitinases can range from 20 to 90 kDa, depending on the source and family classification (Kim et al. 2021). Their pI values may vary from acidic to alkaline, reflecting the wide pH range in which these enzymes can function optimally (Zhang et al. 2018; Churklam and Aunpad 2020). Interestingly, a recent study has reported the extremophilic notoriety of a chitinase derived from a marine autochthonous fungus (Pasqualetti et al. 2022). Understanding the physicochemical characteristics of chitinases is crucial for their purification, characterization, and potential industrial applications. X-ray crystallography and nuclear magnetic resonance (NMR) spectroscopy have provided detailed structural information about chitinases; moreover, a comprehensive list of crystal structures of chitinases and other relevant properties have been deposited in the Protein Data Bank (2019). These studies have revealed the architecture of the active site, substrate-binding clefts, and catalytic residues involved in chitin hydrolysis. The catalytic domain of chitinases adopts a TIM barrel fold, which is essential for their enzymatic activity (Renaud et al. 2023). Structural insights have also enabled researchers to design chitinases with enhanced catalytic efficiency and specificity through protein engineering techniques. For instance, a recent study reported dual glycoside hydrolase 18 (GH18) catalytic domains of a novel chitinase, *Cm*Chi3, which was capable of converting chitin into N-acetly-D-glucosamine as the sole end product (Fig. 4) (Wang et al. 2022). Chitinases hydrolyze chitin by cleaving the β-1,4-glycosidic bonds between N-acetylglucosamine (GlcNAc) units. The catalytic mechanism involves two key subsites in the active site, which accommodate the substrate chain during hydrolysis (Juárez-Hernández et al. 2019). Chitinases are categorized into exo-chitinases and endo-chitinases based on their mode of action. Exochitinases cleave chitooligosaccharides from the non-reducing ends of chitin, whereas endochitinases cleave the polymer chain internally, yielding chitooligosaccharides of various lengths (Churklam and Aunpad 2020; Jiménez-Ortega et al. 2021). Understanding the catalytic mechanism is vital for tailoring chitinases for specific applications. Correspondingly, catalytic efficiency could be extrapolated from their Michaelis-Menten behavior and determined kinetic parameters, such as Michaelis constant (*Km* ) and maximum velocity (*Vmax*). In this regard, chitinases have shown varying affinity (*Km* values) for chitin substrates, indicating their diverse substrate specificities and catalytic efficiencies (Oyeleye and Normi 2018). Chitinases exhibit substrate specificity towards different chitin structures, such as α-chitin, β-chitin, and γ-chitin, which vary in their crystallinity and stability. Some chitinases have shown a preference for specific chitooligosaccharide lengths, suggesting their potential role in specific biological processes (Kidibule et al. 2018; Churklam and Aunpad 2020). Another important indicator for measuring the catalytic performance of chitinases is their behaviour in the presence in the presence of ions and inhibitors, which would ascertain their readiness for industrial and real-world applications on a large scale. Additionally, post-translational modifications and proteolytic processing can regulate the activity of chitinases, making them versatile enzymes with regulatory potential (Singh 2018).

Interestingly, chitinases with enhanced catalytic efficiency and altered substrate specificities have been developed through protein engineering, site-directed mutagenesis, and directed evolution (Akram et al. 2022a, b; Nezhad et al. 2023). For instance, separate reports have revealed that directed evolution and genetic engineering of *Bacillus* and *Vibrio* species improved the chitinase expression and catalytic performance (Wang et al. 2020; Ran et al. 2023; Yuan et al. 2023). These engineered chitinases have found applications in various industries, including agriculture, waste management, biomedicine, and biotechnology.

## Biotechnological exploits of chitinases

**Fig. 5:**
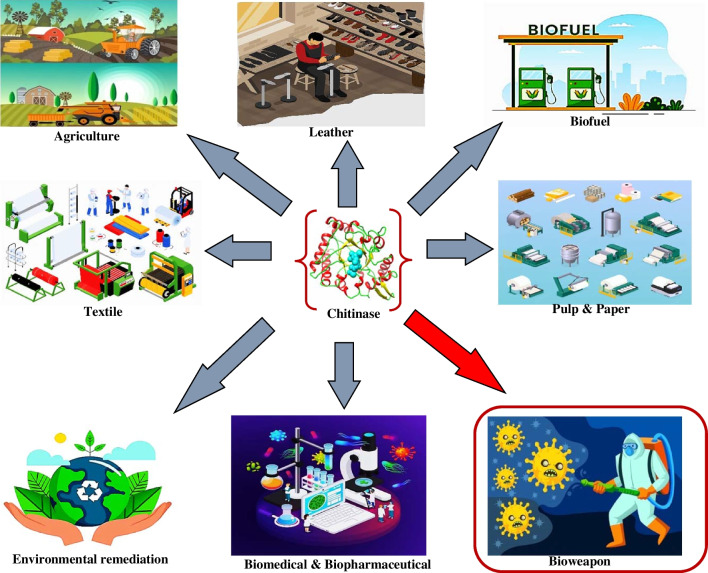
Prospective applications of chitinase (source: this study)

Chitinases have revolutionized bioprocessing by enabling the efficient conversion of chitin-rich waste materials into valuable products. These enzymes facilitate the regioselective depolymerization and transformation of chitin molecules into bespoke chitooligosaccharides, which might serve as precursors to produce bioactive compounds, pharmaceutical intermediates, and functional food ingredients. They further contribute to the development of biodegradable materials and nanoparticles with applications in drug delivery, tissue engineering, and environmental remediation. Chitin-based polymers can be enzymatically modified to create materials with controlled degradation rates and tunable physical properties. Chitinase-mediated production of chitin nanoparticles provides a sustainable alternative to conventional nanoparticle synthesis, although challenges in controlling particle size and stability persist. While bioprocessing with chitinases offers environmental benefits and reduces reliance on petrochemical feedstocks, optimizing enzyme activity, stability, and scalability remains a critical challenge. Moreover, the complexity of chitinase-substrate interactions and the need for precise reaction conditions pose limitations on the scalability and cost-effectiveness of these transformations. The aforementioned biotechnological exploits of chitinases make them relevant in the following industries and processes highlighted infra (Fig. 5).

### Agriculture

**Fig. 6:**
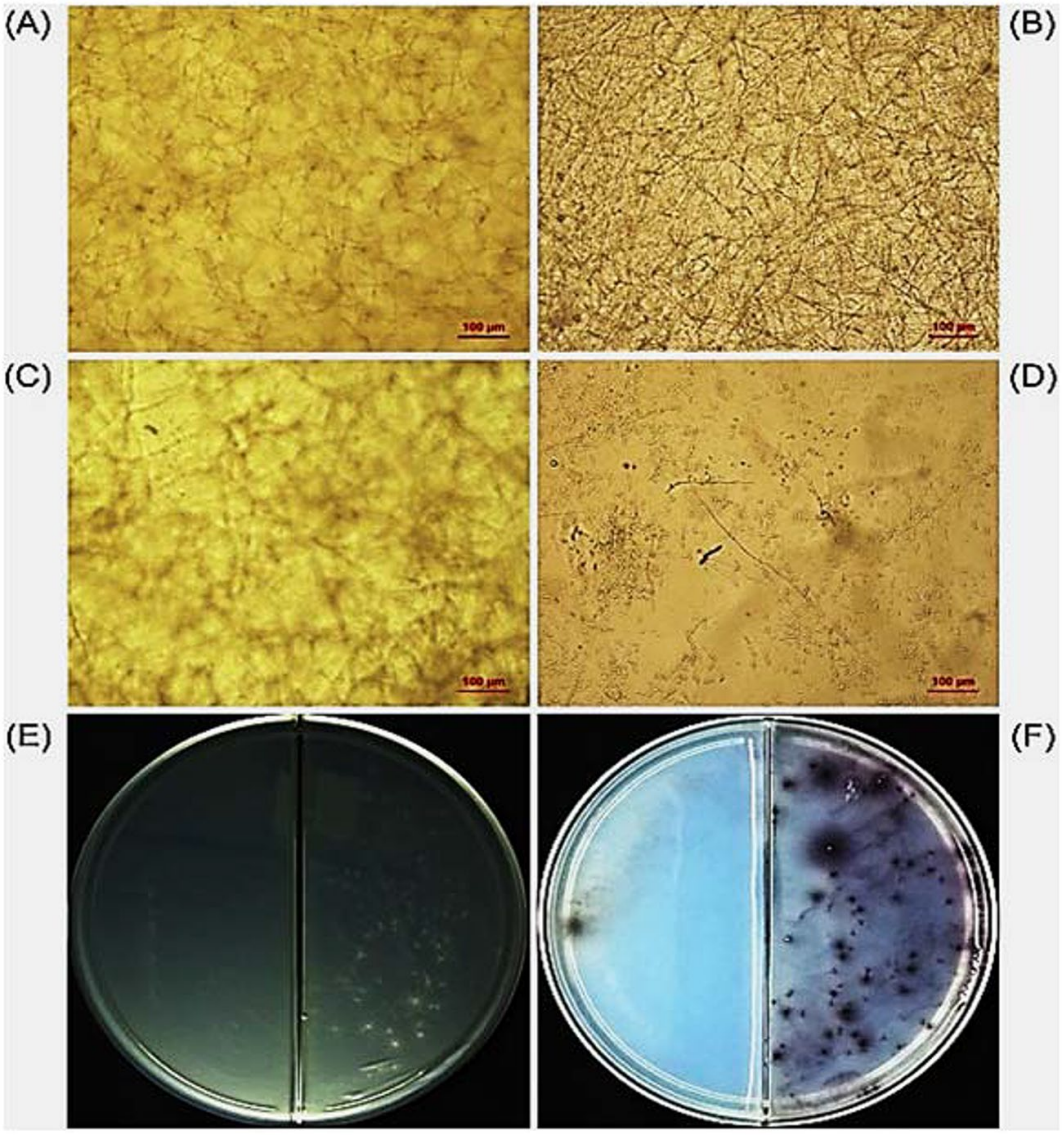
Demonstration of *Cv*Chi47 antagonistic activity against *Fusarium oxysporum* (A, C and E) and *F*. *guttiforme* (B, D and F). (A) Untreated conidia of *F*. *oxysporum*; (B) untreated conidia of *F*. *guttiforme*; (C) conidia of *F*. *oxysporum* treated with *Cv*Chi47 (1 mg/mL); (D) conidia of *F*. *guttiforme* treated with *Cv*Chi47 (1 mg/mL); (E) conidia of *F*. *oxysporum* incubated for 2 h in water (right side of the plate) or in the presence of 1 mg/mL *Cv*Chi47 (left side of the plate) and inoculated onto PDA; (F) conidia of *F*. *guttiforme* incubated for 2 h in water (right side of the plate) or in the presence of 1 mg/mL *Cv*Chi47 (left side of the plate) and inoculated onto PDA. [Reproduced from Sousa et al. (2019) with permission from Elsevier]

Microbial chitinases have gained attention for their potential to enhance various aspects of agriculture. Chitinases play a pivotal role in plant disease management by degrading the chitin-rich cell walls of fungal pathogens (Vaghela et al. 2022). This enzymatic degradation weakens the pathogen’s structural integrity, rendering it more susceptible to host defense mechanisms and chemical treatments. Chitinase-expressing transgenic plants have shown increased resistance to fungal infections (Mahmood et al. 2022). However, the effectiveness of chitinase-based strategies may vary with pathogen species, cell wall composition, environmental conditions, and the timing of enzyme application. For instance, three recombinant chitinases from a *Streptomyces* strain exhibited different rates of growth inhibition against the fungal plant pathogens: *Mucor circinelloides*, *Aureobasidium pullelans*, *Botrytis cinerea* and *Aspergillus fumigatus* (Wang et al. 2023). The antagonistic activity of a thermostable chitinase from *Chromobacterium violaceum* (*Cv*Chi47) on two Fusarium strains is evinced below (Fig. 6) (Sousa et al. 2019).

Chitinases contribute to plant growth promotion by breaking down chitin in the rhizosphere, releasing chitin oligomers that act as signaling molecules. These molecules stimulate plant immune responses, induce systemic resistance, and enhance nutrient uptake (Sharma et al. 2023). Chitinases also aid in the degradation of chitinaceous biofilms, improving nutrient availability and root health (Mehmood et al. 2023). However, the intricate interplay between chitinases, plant hormones, and microbial communities in the rhizosphere requires further elucidation for optimized growth promotion. Chitinases hold promise as biopesticides due to their potential to target chitin-rich pests, such as insects and nematodes. Chitinase-treated formulations have demonstrated insecticidal and nematicidal activity (Rajendran et al. 2023), offering an environmentally friendly alternative to synthetic chemical pesticides. Nonetheless, challenges like specificity and non-target effects necessitate comprehensive ecological impact assessments. Chitinase-mediated induced systemic resistance (ISR) involves the activation of a plant's defense mechanisms against a broad range of pathogens (Ben-Amar et al. 2022; Chouhan et al. 2023). This systemic response is triggered by chitin derivatives released from pathogen cell walls. While promising, the complex signaling pathways and crosstalk involved in ISR induction require detailed exploration to harness their full potential. Microbial chitinases aid in converting chitin-rich waste, such as crustacean shells and fungal biomass, into value-added products useful for soil fertility. For instance, chitinase-driven bioconversion generates chitin oligomers and glucosamine, which possess potential applications as plant growth enhancers, biostimulants, and biopesticides (Khetsha et al. 2022). In summary, chitinase application in agriculture promotes reduced chemical dependency, thereby lessening the burden of detrimental effects on the environment and human health.

### Leather

Leather processing involves depilation, removing hair and fat from animal hides, typically using traditional methods with hazardous chemicals and waste generation. Although efficient leather depilation is prominent amongst hydrolytic enzymes, such as proteases, lipases and keratinases (Khambhaty 2020), chitinases might likewise offer an eco-friendly alternative by breaking down the protein matrix securing hair, enhancing degreasing and dehairing while reducing chemical reliance. Likewise, chitinase could be exploited for efficient collagen extraction from hides rich in collagen fibers, breaking down non-collagenous proteins streamlines extraction, thereby improving leather quality and process efficiency (Lee et al. 2022). Leather processing generates substantial waste like hair, fat, and collagen remnants, which chitinase-driven catalysis might potentially transform into value-added by-products. Chitinase transforms seafood waste, like crustacean shells, into chitosan for leather formulation, enhancing properties like tensile strength and water resistance (Liang et al. 2023). During tanning, chitinase might improve agent penetration by breaking down non-collagenous materials, enhancing texture and softness. Chitinase aids biodegradable, chitosan-based leather finishes, better dye absorption, and environmentally friendly attributes. Chitinase contributes to recycling chitin-containing leather waste, promoting a circular economy by repurposing waste components. Chitinase-driven processes innovate leather production, fostering sustainability and higher quality.

### Biofuel

Chitinase breaks down chitin-rich materials, including fungal cell walls with chitin, enhancing cellulose and lignocellulosic biomass degradation for biofuel production (Giovannoni et al. 2020). It generates biofuel precursors like glucose for microbial fermentation, yielding bioethanol, biobutanol, or other fuels. Agricultural residues and woody materials transform into bioethanol through enzymatic hydrolysis. Moreover, a recent study has evinced the production of bioethanol from chitooligosaccharides, which are derived from chitinolysis (Atheena et al. 2024). As a complementary enzyme, chitinase aids cellulases and hemicellulases, elevating bioethanol efficiency. It optimizes fermentable sugar yields by breaking down chitin-rich segments, enabling diverse feedstock utilization (Kumari et al. 2023). Chitinase enhances biofuel fermentation by acting on chitin-rich inhibitors, aiding microorganisms in higher yields. In algae biorefineries, it breaks down chitin in microalgae cell walls (Brückner and Griehl 2023), boosting lipid and carbohydrate extraction for biodiesel or biohydrocarbons. In anaerobic digestion, chitinase aids chitin-containing waste breakdown, heightening biogas production, mainly methane and carbon dioxide, as a renewable energy source. For microbial fuel production, chitinase-treated chitin-rich feedstocks release fermentable sugars, yielding biogas or biohydrogen. Chitinase treatment of organic waste enhances microbial fermentation, increasing biogas outputs, used directly or processed into biomethane (Bhushan et al. 2023). Fungi inherently producing chitinase aligns with sustainable biofuel production, in line with natural microorganisms' biomass degradation principles.

### Textile

Chitinase might derive relevance in textile processing, particularly in bio-desizing, where it could effectively degrade natural sizing agents and starches on fabrics, easing their removal without harsh chemicals. This enzyme’s role extends to the facilitation of eco-friendly bleaching, aiding color and impurity removal, thereby reducing reliance on chemical bleaching agents and minimizing environmental impact (Biswal and Swain 2023). In the scouring step, chitinase might degrade chitin-based contaminants in natural fibers, yielding cleaner textiles for further processing. Chitinase could also be adopted in fabric softening treatments, breaking down non-cellulosic fabric components for increased softness (Rath et al. 2023). It assists in removing proteinaceous stains, enhancing washing efficacy by breaking down proteins like blood or food stains. In dyeing, chitinase improves dye penetration into textile fibers, achieving vibrant and enduring colors by removing chitin-based barriers. Fabric finishing benefits from chitinase use, eliminating excess chitin and protein-based impurities in natural fibers, resulting in cleaner, smoother, and biodegradable textiles. Chitinase contributes to antimicrobial textiles by degrading chitin in odor or infection-causing microorganisms, suitable for sportswear, healthcare textiles, and specialized products (da Silva et al. 2023). In dyeing preparation, chitinase removes impurities affecting dye uptake and color fastness, enhancing dyeing efficiency and quality. Chitinase-based finishing processes might enhance textile properties like antimicrobial or moisture-wicking characteristics, aligning with the demand for sustainable textile treatments.

### Paper

Chitinase might serve as a bio-bleaching agent in the paper industry, aiding lignin and impurity removal from pulp for brighter, higher-quality paper. Chitinase-derived chitosan acts as a bio-based papermaking additive, enhancing strength, water retention, and sustainability (Prasetiyo et al. 2021). In paper manufacturing, chitinase addresses wastewater pollutants by breaking down chitin-containing substances, reducing effluent’s environmental impact. Chitinase facilitates and optimizes the deinking process for recycled paper by breaking down ink and impurities, enhancing efficiency and paper quality (Farkas et al. 2020). Chitinase aids paper recycling by degrading coatings, adhesives, and fiber contaminants, elevating deinking efficiency and recycled paper quality. It might mitigate pitch problems in wood pulp by breaking down chitin-like components, improving processing. Chitinase-based coatings enhance paper biodegradability, which is crucial as the industry seeks eco-friendly alternatives. Chitinase treatment boosts paper-based packaging biodegradability by degrading chitin-rich components, reducing waste. Antimicrobial properties emerge in chitinase-treated paper due to microbial-supporting chitin-rich component degradation, valuable for packaging and hygiene products (Priyadarshi and Rhim 2020). Chitinase could improve pulping efficiency by breaking down chitin-containing raw material components, enhancing fiber separation and lowering energy consumption.

### Environmental remediation

Chitinase enhances bioremediation at contaminated sites by breaking down chitin-rich pollutants into less toxic compounds, aiding microbial degradation (Akram et al. 2022b), which reduces harmful substances in soil and water, promoting environmental health. Chitin-based materials might absorb heavy metals harmful to ecosystems and health, which might be difficult to detect and remove; here, chitinase, being participatory in heavy metal resistance could degrade metal-bound chitin, releasing metals for easy access and removal. It could also facilitate the breakdown of chitin-rich biofilms in water and wastewater reticulation systems, preventing accumulation; in contaminated aquatic environments, it could improve water quality and maintain ecological balance. Chitinase degrades chitin-containing components of plastics, reducing plastic waste’s impact. It addresses microplastic pollution by breaking down chitin matrices, and releasing trapped microplastics (Zhou et al. 2022). Chitinase improves wastewater treatment by breaking down chitin-based materials and organic matter, enhancing treatment efficiency. Chitinase’s role in nutrient cycling releases plant-available nutrients from chitin-rich materials, supporting ecosystem health; during phytoremediation, it modifies plants to degrade chitin-based pollutants. It might also target invasive species and harmful algal blooms in aquatic environments, controlling their proliferation (Coyne et al. 2022). Although renowned as a biopesticide, chitinase might further break down synthetic pesticide residues with structures analogous to chitin, thereby minimizing environmental impact. It could also prove resourceful in oil spill cleanups, by enhancing oil dispersant effectiveness (Song et al. 2022). Chitinase accelerates land restoration by breaking down chitin-containing matter, thereby improving soil health, nutrient cycling, and microbial activity. It might likewise act as a bio-indicator in monitoring organic matter degradation and ecosystem health and could enhance microbial fuel cells using chitin-rich waste for energy production and remediation through the breakdown of chitinous wastes from textile and paper industries, thereby reducing pollution.

### Biomedical and pharmaceutical

Chitinase-based therapies enhance wound healing and tissue regeneration by breaking down chitin-rich components in wound dressings, promoting necrotic tissue removal (Mathew et al. 2021b). It modifies chitin-based scaffolds for tissue engineering; in joint tissues affected by osteoarthritis, chitinase targets chitin-containing components, reducing inflammation (Madan et al. 2020). It might also facilitate bone scaffold modification, enhancing biocompatibility and bone tissue regeneration (Desai et al. 2023). Chitinase’s role in cancer treatment disrupts chitin-containing components in tumor microenvironments, potentially affecting tumor growth. In drug delivery, chitinase modifies chitin-based carriers, improving drug release and targeted delivery as well as drug efficacy (Mahajan et al. 2023). It targets bacterial and fungal cell walls as antimicrobial agents, inhibiting growth, and thereby enhancing antifungal drug effectiveness. Chitinase could manage gastrointestinal disorders by modulating chitin-containing components, thereby enhancing nutrient bioavailability by modifying chitinous dietary supplements or pharmaceuticals (Tabata et al. 2019). Chitinases possess anti-inflammatory and immunomodulatory effects, used in therapies for inflammatory diseases (Viana et al. 2017). In oral healthcare, chitinase (present in saliva) targets pathogenic yeasts and their biofilms in cavities, aiding in preventing dental issues (Amerongen and Veerman 2002). Chitinase enhances vaccine responses, developing biocompatible materials for medical devices. Chitinase’s presence in bodily fluids serves as a disease biomarker. For instance, Tabata et al. (2017) identified the high levels of chitotriosidase in the plasma of patients affected by Gaucher’s disease. Likewise, high levels of CHI3L1 have been documented in patients with inflammatory conditions such as Crohn’s disease, ulcerative colitis, asthma and serum liver cirrhosis (Kušnierová et al. 2020).

### Biological warfare

**Fig. 7:**
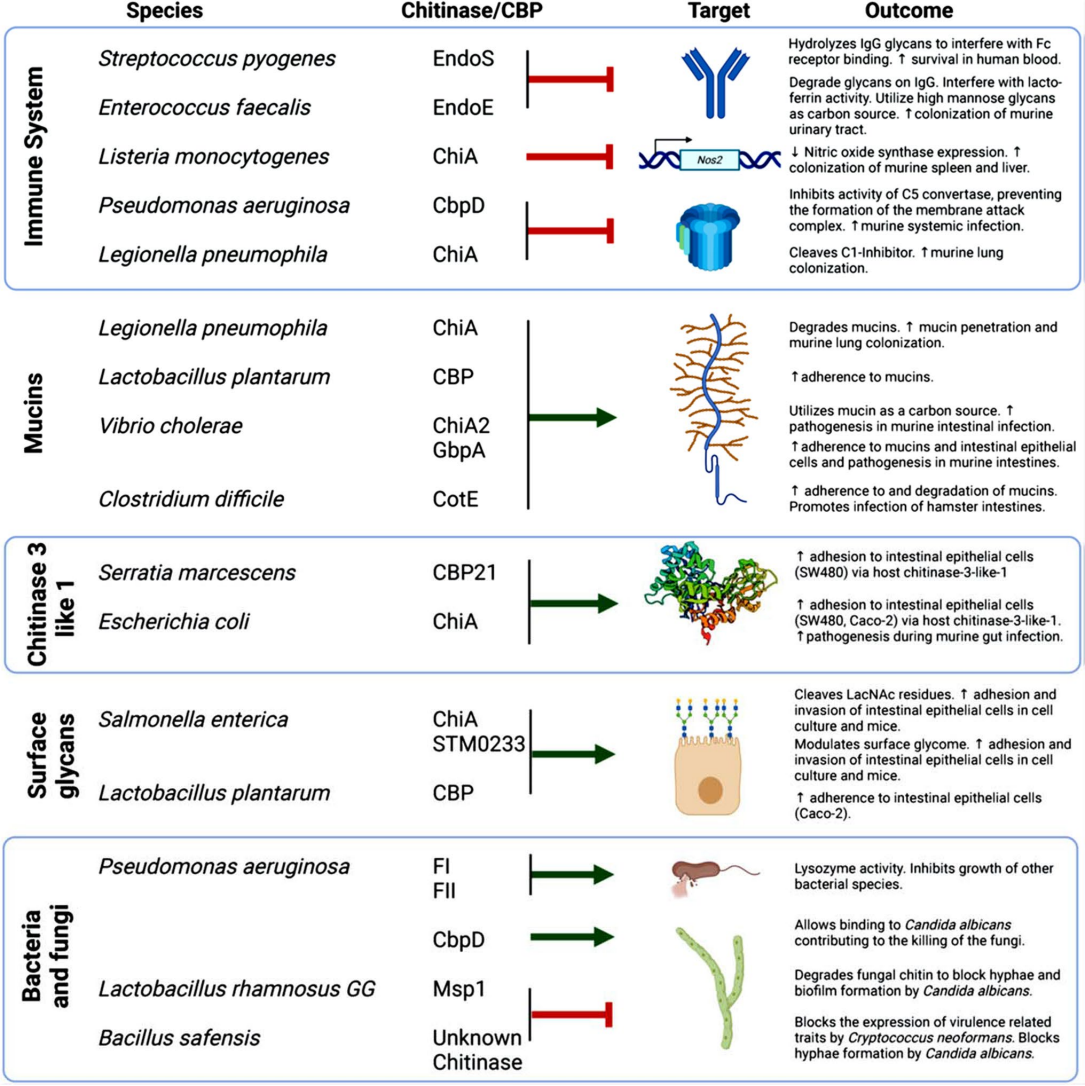
The interaction of bacterial chitinases/chitin binding proteins (CBPs) with molecular targets that are relevant to human and mammalian infection. (Source: Devlin and Behnsen (2023) copyright © American Society for Microbiology Infect Immun 91(7): e00549-22

Chitinase’s potential risks in biological warfare must be considered in the broader context of biosecurity. This is because advances in biotechnology could inadvertently facilitate harmful uses of chitinase, accentuating the ethical and biosecurity concerns that arise due to chitinase's dual-use nature. Essentially, chitinases, once released, could have the potential to uncontrollably spread in the environment, affecting both intended targets and non-target organisms, as long as they are chitinous in nature (Singh and Arya 2019). Unlike traditional chemical agents, chitinases are biologically active and may continue to degrade chitin-rich materials even after the initial deployment, potentially disrupting ecosystems for an extended period. This is highly realizable due to the ability of chitinase to be stable for extended periods of time, as corroborated by a study where chitinase still possessed appreciable quotients of activity after 2 months of storage (Cheba and Zaghloul 2020) Chitinase’s capacity to degrade insect and fungal structures raises worries about the misuse of its technology. Insects and fungi reproduce rapidly, potentially allowing engineered chitinases to propagate and amplify their effects at an accelerated pace. While chitinase can break down chitin, its malicious application could harm ecosystems, essential crops, and food chains, posing threats to agriculture and the environment. The release of chitinase could cause widespread food shortages and economic disruption, impacting agriculture and food security (Fiorin et al. 2018). Chitinase-based bioweapons might target chitin-dependent organisms, affecting biodiversity, leading to ecological imbalances and economic instability, and might likewise pose challenges for recovery or restoration due to complex ecological interactions. Chitinase’s potential use in biological warfare might adversely impact infrastructure, human health, and ecosystems. For instance, chitinase-producing agents could be designed to degrade chitin-rich materials found in various infrastructure components, such as wood, concrete, and textiles, leading to the deterioration of critical infrastructure, including buildings, artefacts, bridges, and transportation systems, causing significant economic and social disruptions. Although chitin is not a major component of human tissues, some chitinases may have unintended effects on human cells or induce allergic reactions and immune responses (Leoni et al. 2019; Chandra et al. 2022). The potential for allergic reactions or immune-mediated diseases raises concerns about their impact on civilian populations. For instance, a recent review by Devlin and Behnsen (2023) highlighted chitinase’s status as a potent virulence factor in WHO critically ranked bacterial pathogens and further discussed its role during gastrointestinal, respiratory and systemic infections (Fig. 7). Interestingly, the role of chitinase and chitinase-like proteins in the epidemiology of pediatric lung diseases has been discussed, where increased levels of YKL-40, a chitinase-like protein was associated with severe asthma, cystic fibrosis and other inflammatory disease conditions (Mack et al. 2015). In another study, the significance of chitinase-3-like protein 1 (CHI3L1) as a marker of disease diagnosis, prognosis, activity and severity was appraised. It was further elucidated that the enzyme was upregulated by various inflammatory and immunological diseases, including several cancers, Alzheimer’s disease, and atherosclerosis (Yu et al 2024). Chitinase’s deployment in biological warfare could have global implications, affecting regions through environmental contamination and public health risks. Over time, the indiscriminate use of chitinase-based biological weapons could lead to the evolution of chitinase-resistant pathogens or organisms (Tully and Huntley 2020). This could make controlling disease outbreaks more challenging and potentially render extant prophylaxis ineffective. Chitinases could facilitate the penetration of pathogens into host organisms, increasing the severity and lethality of infections (Krone et al. 2023). The difficulty in attributing attacks complicates response efforts; this underscores the complexity of addressing chitinase-based threats. Moreover, detecting and mitigating chitinase-based attacks would be time and resource-intensive. Chitinase’s misuse raises questions about responsible science and effective oversight. The dual-use nature of chitinase complicates monitoring and regulation and therefore underscores the importance of responsible research, ethical considerations, and stringent regulation to prevent its malicious application.

## Commentary

Chitinases, a class of enzymes capable of degrading chitin, play a significant role in various biological processes, offering both potential benefits and inherent risks when applied in diverse industries. Derived from a range of sources, including microorganisms, plants, and animals, chitinases exhibit unique characteristics that make them versatile tools for industrial applications. However, their use presents challenges related to regulation, ethical concerns, and unintended ecological consequences. Chitinases possess the remarkable ability to break down chitin, a complex polymer that forms the structural component of fungal cell walls, arthropod exoskeletons, and other chitin-containing materials. This unique enzymatic activity stems from their active sites, which cleave the glycosidic bonds in chitin molecules, ultimately leading to its degradation into simpler compounds. This property makes chitinases invaluable for various applications in industries like agriculture, waste management, textile, leather, biotechnology, and medicine. For instance, in agriculture, chitinases find utility in combating pests and diseases. Their incorporation into genetically modified crops confers resistance to insects that rely on chitin-based exoskeletons. Furthermore, chitinase-treated agricultural waste can be converted into value-added products like biofuels or fertilizers, contributing to sustainable resource management. In medicine, chitinases exhibit promise in wound healing, tissue engineering, and drug delivery, presenting opportunities for improved healthcare and therapeutic interventions. Despite their potential benefits, the industrial application of chitinases is not without challenges. One key hurdle is regulatory oversight. Chitinases’ dual-use nature, where their beneficial applications can be repurposed for harmful purposes, necessitates careful monitoring and control to prevent misuse, particularly in bioweapon development. Ethical considerations arise as well, especially when chitinases are employed in ways that may disrupt ecosystems or unintended species, potentially leading to ecological imbalances. Chitinase applications can also yield unintended ecological consequences. Their widespread use may inadvertently affect non-target organisms, disrupt food chains, and alter ecosystems, underlining the importance of thorough risk assessment and responsible research practices. Moreover, there are concerns about chitinase resistance developing in pathogens, rendering treatments ineffective and exacerbating disease outbreaks. Therefore, to harness the benefits of chitinases while minimizing the associated risks, a balanced approach involving rigorous oversight, responsible research, and comprehensive risk assessment is essential.

## Data Availability

Sources of data collected have been mentioned in the text.
